# Long read, isoform aware sequencing of mouse nucleus accumbens after chronic cocaine treatment

**DOI:** 10.1038/s41598-021-86068-7

**Published:** 2021-03-24

**Authors:** Molly Estill, Efrain Ribeiro, Nancy J. Francoeur, Melissa L. Smith, Robert Sebra, Szu-Ying Yeh, Ashley M. Cunningham, Eric J. Nestler, Li Shen

**Affiliations:** 1 Nash Family Department of Neuroscience and Friedman Brain Institute, New York, USA; 2 Department of Genetics and Genomic Sciences and Icahn Institute for Data Science and Genomics Technology, Icahn School of Medicine At Mount Sinai, New York, NY 10029 USA; 3 Sema4, A Mount Sinai venture, Stamford, CT USA

**Keywords:** Addiction, Brain, Transcriptomics

## Abstract

To better understand the full-length transcriptome of the nucleus accumbens (NAc)—a key brain reward region—in chronic cocaine treatment, we perform the first single molecule, long-read sequencing analysis using the Iso-seq method to detect 42,114 unique transcripts from mouse NAc polyadenylated RNA. Using GENCODE annotation as a reference, we find that over half of the Iso-seq derived transcripts are annotated, while 46% of them harbor novel splicing events in known genes; around 1% of them correspond to other types of novel transcripts, such as fusion, antisense and intergenic. Approximately 34% of the novel transcripts are matched with a compiled transcriptome assembled from published short-read data from various tissues, with the remaining 69% being unique to NAc. These data provide a more complete picture of the NAc transcriptome than existing annotations and can serve as a comprehensive reference for future transcriptomic analyses of this important brain reward region.

## Introduction

The brain’s reward system is a key driver of the response to intrinsic and extrinsic rewarding stimuli^1^. In mammals, the brain’s dopaminergic pathways, which include the mesocorticolimbic pathway, are known to be disrupted by addiction. This mesocorticolimbic pathway, with a well-established role in pleasure and reward, originates in the ventral tegmental area (VTA) and projects to several forebrain regions, including the nucleus accumbens (NAc), amygdala, and hippocampus^1^. The long-lasting effects of drugs of abuse on this reward circuitry are suspected to be mediated by epigenetic changes, such as chromatin accessibility, and by alterations in gene expression^2^.

In rat models, cocaine injections have been shown to cause gene “priming” of the *FosB* gene in the NAc, allowing for rapid transcription of *FosB* after a cocaine challenge^3^. In a murine model of cocaine addiction and relapse, genes associated with the first-ever exposure to cocaine and chronic cocaine administration across PFC, dorsal striatum (DStr), NAc, basolateral amygdala (BLA), ventral hippocampus (vHIP), and VTA were identified^4^. Due to the crucial role of the NAc in mediating addiction, a thorough characterization of the chromatin and transcriptomic landscape is needed to understand the changes induced by addictive substances^4,5^. However, all studies to date have relied on short read RNA-seq data^6^ to characterize the transcriptome, which is excellent for gene expression quantification but falls short in its ability to identify full-length transcripts with isoform resolution. Although alternative splicing induced by drug treatment was described in previous studies^7–10^, these works were based on previously annotated transcriptomes. Here, we utilized Single Molecule, Real-Time (SMRT) sequencing, combined with Iso-seq methods^11^, to characterize the polyadenylated transcriptome of the mouse NAc under chronic cocaine and saline administration conditions to provide a more comprehensive picture of the transcriptome of this brain reward region. The novel transcripts were further examined using short-read RNA-seq data from a previous study^10^ performed in the mouse NAc to assess short-read support of the full-length transcripts. As far as we know, this is the first application of Iso-seq analysis to in vivo brain tissue from a drug addiction study.

## Results

### Classification of long-read isoforms

The objective of this study was to identify the NAc transcriptome, as well as any splicing variants that may be induced or repressed by cocaine exposure. To accomplish this, male C57BL/6 J mice were injected with saline or cocaine (5 in each group) for 7 days. As the focus of this study was on robustly identifying transcripts in a cost-effective manner, the NAc tissues for all animals were pooled. 3,145,365 full-length (FL) reads were obtained and after de novo isoform prediction using the IsoSeq3.1 pipeline (SMRTLink, v8.0), were subsequently collapsed to 199,974 high-quality reads. 99.9% of all high-quality reads (199,879 reads) were successfully aligned to the mm10 genome. An initial set of 55,310 unique isoforms were identified, which were classified and filtered using SQANTI2^12,13^ for a final set of 42,114 unique isoforms. SQANTI2^12–14^ was used to provide a detailed classification of the transcripts, in comparison to the established GENCODE annotation (release M25)^15^. The SQANTI2 classifications, which include eight categories, are described briefly here. “Full Splice Match” (FSM) and “Incomplete Splice Match” (ISM) are isoforms that match reference transcripts at all splice junctions (SJs) or only a portion of the consecutive SJs, respectively; these two categories are not considered to be novel. The remaining six classes—Intergenic, Genic Genomic, Antisense, Fusion, Novel in Catalog (NIC) and Novel Not in Catalog (NNIC)—are considered to be novel (for examples, see Supplementary Fig. S1). Specifically, novel rearrangements of known or unannotated SJs are classified as NIC and NNIC, with NIC transcripts representing new combinations of previously annotated donors and acceptors, while NNIC transcripts include novel donors and/or acceptors. The remaining four novel categories represent isoforms that have no exon congruency with known annotations. The category “Genic Genomic” represents those transcripts with partial exon overlap in a known gene, but do not share known SJs. “Intergenic” transcripts lie completely outside annotated genes. “Antisense” transcripts are polyadenylated transcripts overlapping the complementary strand of an annotated transcript, while “Fusion” indicates a transcript joined from two independently annotated loci. We also compared results derived from the SQANTI2 tool with another commonly used pipeline, MatchAnnot^16^, and found that they overlapped very well, but SQANTI2 provided a more detailed annotation (Supplementary Fig. S2 and Supplementary File 1). This level of fine categorization can be important for distinguishing the potential roles of a novel transcript, particularly for genes that have many different isoforms.

**Figure 1:**
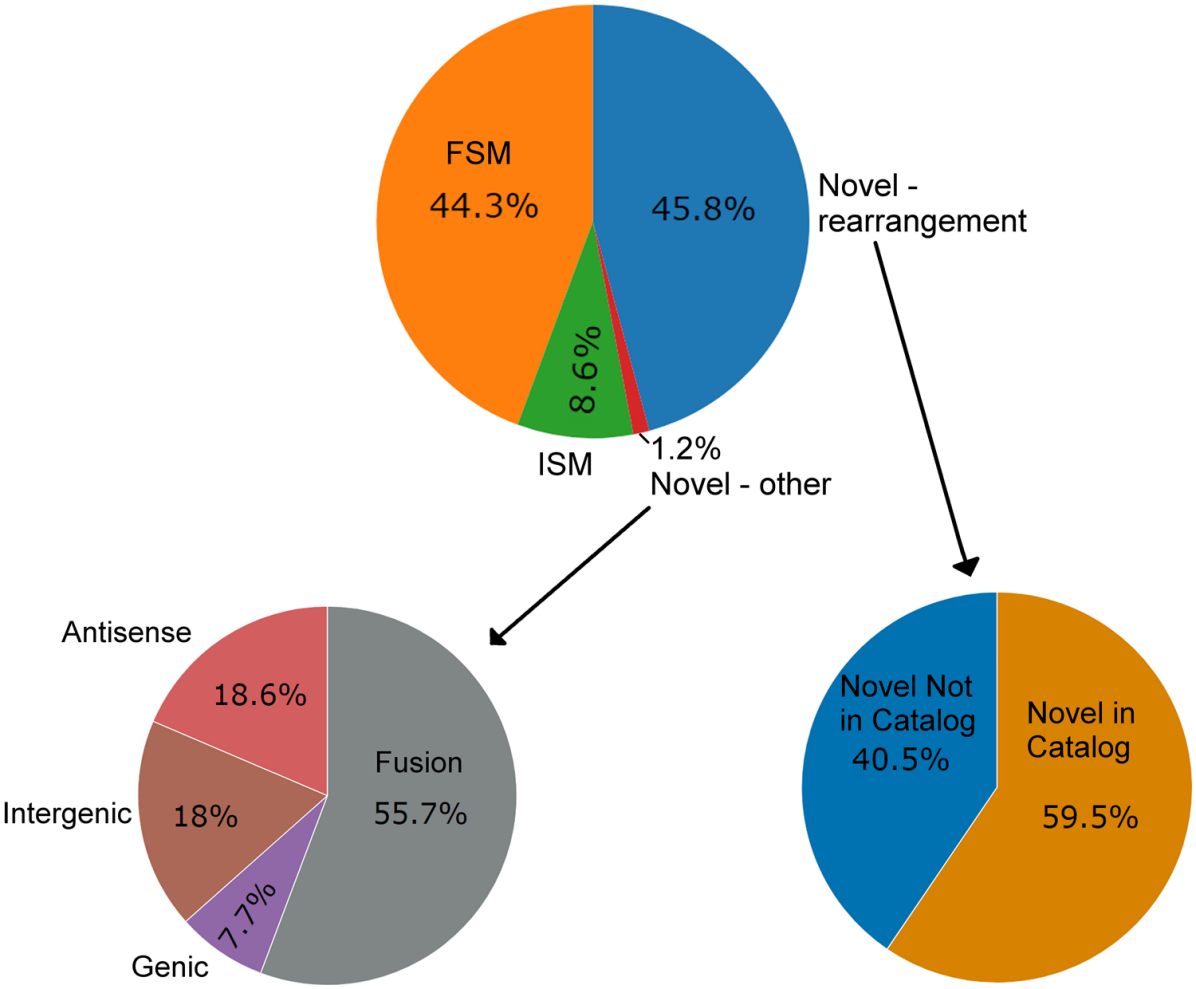
Distribution of SQANTI2 classifications of Iso-seq transcripts. The novel isoform categories are grouped into “Novel-rearrangement” and “Novel-other”. The “Novel-rearrangement” represents SJ rearrangements (NIC and NNIC). The “Novel-other” represents all four remaining novel categories (Genic genomic, Intergenic, Antisense and Fusion).

Based on the SQANTI2 classification (Fig. 1), 53% of the 42,114 Iso-seq derived transcripts were classified as previously known, with 44% being FSM and 9% being ISM. The rest were largely classified as novel SJ rearrangements of known genes (i.e., NIC and NNIC), which altogether comprised almost 46% of the isoforms. The remaining novel categories (Genic Genomic, Intergenic, Antisense and Fusion) together comprised 1.2% of the isoforms. The smallest novel categories are Intergenic and Genic Genomic, which comprised only 0.2% and 0.01% of the isoforms, respectively.

The vast majority of the SJs utilized in the mammalian transcriptome comprise the canonical nucleotide compositions of GT-AG, GC-AG and AT-AC at the beginning and the end of the intron, with GT-AG being the dominant form. Altogether, these canonical compositions encompass the vast majority (> 99%) of the human SJs^12–14^. As expected, the majority of the Iso-seq transcripts in our dataset used known canonical SJs (91.8% of SJs), with only 0.04% of SJs being known non-canonical. The remaining 8.1% (n = 11,110) of SJs were not present in the GENCODE annotation and were thus considered to be novel. All identified novel SJs were classified as “novel canonical”, indicating that the SJs had not been previously annotated, but utilized canonical nucleotide compositions.

It should be noted that each transcript assessed in this study is supported by two or more FL Iso-seq reads, which is expected to reduce the incidence of false positives in this analysis^17^. The veracity of the novel isoforms was also assessed through an overlap with publicly available Cap Analysis Gene Expression (CAGE) peaks, collected across a variety of murine primary cells and cell lines^18^. The CAGE peaks used in this study consisted of prior known CAGE peaks (remapped to mm10) and newly identified CAGE peaks in the FANTOM5 release^18,19^. As shown in Table 1, the novel categories show an excellent overlap with the CAGE peaks. Even in the case of intergenic transcripts, which have the lowest overlap with the CAGE peaks, 46% of the transcripts overlap a CAGE peak. Additionally, CAGE analysis, which is designed to capture the 5′ ends of capped mRNAs, can only measure transcriptional initiation frequencies for the tissue it was performed in. Therefore, the lack of overlap by some transcripts may be due to the lack of CAGE analysis in the mouse NAc tissues. In total, 17 exon-exon junctions defining novel transcripts (6 intergenic, 3 fusion, 4 genic, and 4 antisense) were verified by both qRT-PCR and Sanger sequencing (Supplementary File 2 and Supplementary Fig. S3).

**Table 1:** CAGE overlap and coding prediction of the novel transcripts break down into the eight categories.

	NO CAGE overlap		CAGE overlap	
	Coding	Non-coding	Coding	Non-coding
Antisense	10.6% (10)	20.2% (19)	18.1% (17)	51.1% (48)
Intergenic	1.1% (1)	52.7% (48)	3.3% (3)	42.9% (39)
Fusion	5% (14)	2.5% (7)	84.4% (238)	8.2% (23)
Genic	7.7% (3)	15.4% (6)	41% (16)	35.9% (14)
FSM	3% (551)	1.2% (217)	92% (17,175)	3.9% (728)
ISM	19.9% (724)	1.3% (48)	76% (2768)	2.7% (100)
NIC	3% (350)	0.2% (23)	95.2% (10,923)	1.6% (182)
NNIC	6.2% (488)	1% (81)	88.7% (6934)	4% (310)

For each row, the proportions and the corresponding counts of each category are shown.

### Comparison of the NAc transcriptome to previous mouse annotation

Previous studies have made available a series of transcriptomes from a variety of mouse tissues using both short- and long-read sequencing. A compiled transcriptome was derived from three short-read based studies—the first study^20^ (GSE125483) includes a variety of mouse tissues (Adrenal, Colon, Heart, Liver, Lung, Muscle, Pituitary, Skin, Thyroid and Brain); the second study^21^ (GSE107423) includes healthy brain tissues; and the third study^22^ (GSE112348) includes a specific brain region (Cortex)—and two long-read based studies—the first study^23^ includes various murine tissues (including brain) (GSE93848) and the second study^24^ includes preimplantation embryos (GSE138760). In addition, the NONCODE database for mouse^25,26^, which contains a reference of long non-coding transcripts, was added to the compiled transcriptome (Supplementary File 3).

Therefore, the compiled data represented a comprehensive collection of mouse transcriptomes with an emphasis on brain-specific transcripts. Notably, the compiled transcriptome includes transcripts from multiple brain regions, including NAc. However, due to the small size of NAc (in comparison to the whole brain), the contribution of the NAc-specific transcripts to the transcriptome was assumed to be negligible. Therefore, these external studies can be used to complement the GENCODE annotation and help us better distinguish whether an Iso-seq derived transcript has expression evidence in other murine tissues. We will refer to the compiled transcriptome as the “brain-enriched whole mouse transcriptome” (BWMT). The Iso-seq derived transcripts were compared to the BWMT using SQANTI2^12–14^ (Supplementary File 4). If an Iso-seq transcript was classified as novel by GENCODE, but as FSM or ISM using the BWMT as a reference, it was considered to be shared between the NAc and other mouse tissues or “BWMT-matched”; if a transcript was classified as novel by both GENCODE and BWMT, it was determined to be “NAc-specific”; the transcripts that were classified as FSM or ISM by GENCODE were ignored here since they were already annotated.

**Figure 2:**
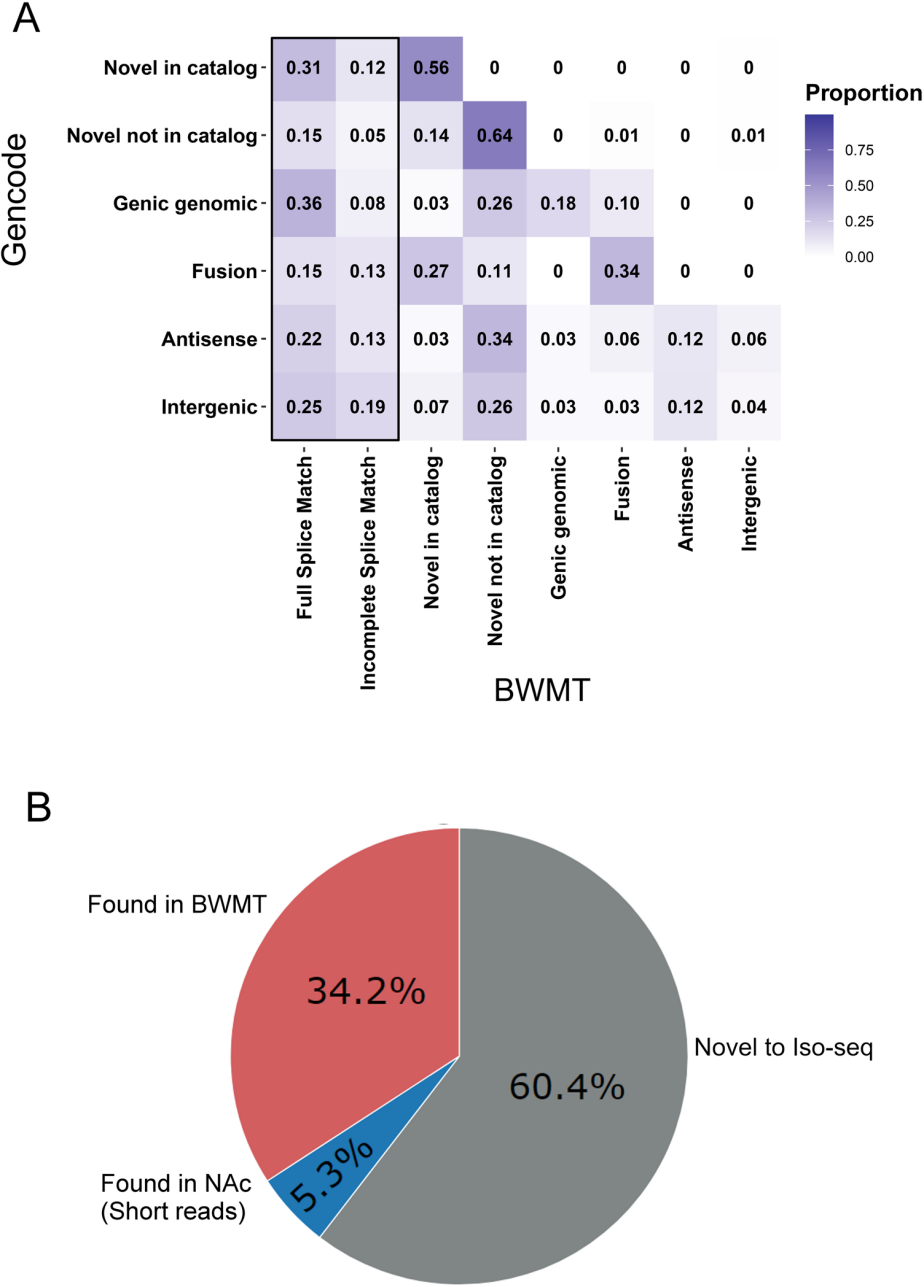
Comparison of Iso-seq NAc transcripts to a published murine annotation. (**A**) Confusion matrix of Iso-seq transcript classifications, based on the novel transcript assignments in the GENCODE annotation and the BWMT annotation. For each matrix row, a cell’s value is presented as a proportion of the row’s sum. Novel transcripts with a complete match (FSM or ISM) in the BWMT reference are highlighted with a black rectangle. (**B**) Distribution of novel Iso-seq transcripts in NAc, based on the transcript assignments in the BWMT annotation and the de novo NAc annotation. Novel transcripts are defined as being classified other than FSM or ISM. 6764 of the 19,788 transcripts identified as novel according to the GENCODE annotation overlap the FSM and ISM transcripts in the BWMT annotation. The remaining 13,024 transcripts are considered NAc-specific, with 1064 transcripts being identified as FSM or ISM in the de novo NAc annotation.

We performed a head-to-head comparison of the transcript classifications between the two transcriptomes using a confusion matrix (Fig. 2A). Of particular interest were the transcripts that were classified as one of the novel categories (NIC, NNIC, Genic Genomic, Fusion, Antisense or Intergenic) using the GENCODE reference. As shown in Fig. 2A, approximately 46% and 17%, respectively, of the NIC and NNIC GENCODE classifications were re-assigned as FSM or ISM in the BWMT reference, that is, the transcripts were BWMT-matched. Approximately 44% and 28% of the Genic Genomic and Fusion categories, respectively, were found to be a BWMT match. Similarly, approximately 35% and 44% of the Antisense and Intergenic categories, respectively, were a BWMT match. Surprisingly, a total of 96% of the Intergenic classifications were re-assigned as a non-intergenic category in the BWMT reference, suggesting many “intergenic” transcripts were merely missed records in the GENCODE annotation. Altogether, this comparison suggests that the BWMT reference can complement the GENCODE to more accurately classify the Iso-seq derived transcripts.

We previously have examined the transcriptomes of mouse NAc after chronic saline and cocaine exposure via short-read (100 bp single-end) RNA-seq^10^. To determine if the NAc-specific transcripts identified in this study can be assembled from short-read NAc sequencing, we used SPADES^27^ to generate a de novo transcriptome from the short-read data (Supplementary File 5). As shown in Fig. 2B, only 8.2% of the NAc-specific transcripts were matched in the de novo short-read NAc transcriptome. This shows that the Iso-seq long-read sequencing is a powerful tool to discover previously unknown transcripts.

### Expression analysis of novel and known transcripts using short read data

Although the Iso-seq technique excels at elucidating the full-length transcriptome and identifying novel variants, it cannot provide sufficient sequencing depth for gene expression quantification with a reasonable cost. To address this limitation, we integrated the short-read data from mouse NAc with the Iso-seq derived transcripts from the present study. Towards this end, replicates of both cocaine and saline (3 replicates in each condition) short-read libraries were processed with a pseudo-aligner, Kallisto^28^, to the Iso-seq reference transcriptome. Isoform expressions were normalized as transcripts per million (TPM). Genes expected to be highly expressed in brain were verified to be also highly expressed using the short-read libraries. For example, *Gfap* (encoding glial fibrillary acidic protein), a major filament protein of astrocytes, is highly expressed in human^29^ and mouse^30^ brain tissue and the major isoforms of *Gfap* showed high expressions in both short-read and Iso-seq long-read sequencing libraries (Supplementary Fig. S4).

Among the different SQANTI2 categories, certain categories, such as FSM and ISM, were associated with slightly higher expression values than the others (Supplementary Fig. S5). This was expected due to the associations of these categories with well-annotated genes. However, it was interesting that the “intergenic” category had a large expression range, suggesting that transcripts derived from intergenic regions may be heavily expressed despite originating from previously thought non-genic stretches of the genome. Ontological analysis of genes at the top 10% TPM value revealed the most significant enrichment in the Mouse Gene Atlas database to be NAc (N = 205; adjusted *p*-value = 5.8e-64), which was expected.

Among the novel transcript categories, NNIC and NIC had the largest gene lists. The enrichment of these gene lists in ontological databases and murine tissues revealed that NNIC genes were significantly associated with several cellular components, including cytosol (N = 644; adjusted *p*-value = 5.3e-12), Golgi apparatus (N = 244; adjusted *p*-value = 9.7e-8), and synapse (N = 127; adjusted *p*-value = 3.6e-7). NIC genes were significantly associated with cytosol (N = 843; adjusted *p*-value = 1.7e-34), nucleolus (N = 560; adjusted *p*-value = 1.4e-23), and mitochondrion (N = 446; adjusted *p*-value = 5.1e-22). When examining the enrichments in biological functions, both NNIC and NIC were most significantly associated with regulation of GTP (NNIC: N = 157, adjusted *p*-value = 2.1e-8; NIC: N = 185, adjusted *p*-value = 6.9e-11) (Supplementary File 6). In neurons, GTP cycling plays a major role in proper neuronal functioning, including the synaptic terminal and neurotransmitter release^31,32^. Such genes associated with GTP cycling include SYNGAP1, whose protein is a Ras GTPase activating protein, and which is essential for normal postsynaptic signaling. Additionally, mutations in SYNGAP1 are known to cause intellectual disability in humans^33^. Notably, the NNIC category is highly associated with regulation of synaptic plasticity (N = 51; adjusted *p*-value = 1.3e-5). This includes genes involved in fusion of vesicles to the synapse, such as SNAP25, a component of the SNARE complex^34^, and SYP, encoding synaptophysin, an integral membrane protein of synaptic vesicles^35^. Alternative splicing has been extensively implicated in neuronal development, function and synaptic plasticity^36,37^. For example, neurexin, a class of synaptic adhesion molecules, are known to have thousands of distinct isoforms in mice^36^.

We searched known addiction-related genes for the presence of novel transcripts in our data. Genes identified in a previous study as having a positive or negative association with cocaine addiction in mice across several brain regions were examined for evidence of novel transcripts^4^. While the majority of the examined genes were not associated with novel transcripts in the Iso-seq dataset, several interesting novel transcripts were identified. FGF10, a fibroblast growth factor involved in cell growth, is expressed across a range of murine tissues. In this study, a single-exon transcript antisense to the 5′ end of the *Fgf10* gene, PB.12758.1, was identified. Steroid receptor RNA activator 1 (SRA1) is negatively associated with cocaine addiction^4^, and its gene is located downstream of the gene encoding Amyloid Beta Precursor Protein Binding Family B Member 3 (APBB3). Two novel transcripts, PB.15617.1 and PB.15617.2, were found to be fusion transcripts of *Apbb3* and *Sra1*. This phenomenon appears to be caused by a read-through of *Apbb3* into its downstream neighboring gene, *Sra1*. Both fusion transcripts were predicted to encode proteins, with coding probability scores of 0.994 and 0.988, respectively. This read-through product was also matched with several similar read-through transcripts in the BWMT transcriptome.

FosB, a transcription factor implicated in the neuronal response to stress and drugs^38,39^, as well as its splice variant, ∆FosB, show evidence of a 5′ truncation due to an alternative transcription start site (TSS)^40^. The novel transcripts PB.6409.1 and PB.6409.2 lack the first exon of *FosB* and *∆FosB*, implying the presence of multiple TSSs. While this study did not validate the novel 5′ truncations of *FosB* or *∆FosB* with targeted PCR or Sanger sequencing, it should be noted that the BWMT transcriptome also contains evidence for these 5′ truncated transcripts. Additionally, the FANTOM5 CAGE dataset overlaps the pre-established first exon of the novel, truncated transcripts.

Lastly, a Y-linked gene associated with the NAc in male mice^41^, encoding EIF2S3Y (eukaryotic translation initiation factor 2 subunit 3 y-linked), was found to have three novel splice variants in both truncated and full-length forms of the transcript (PB.16991.1, PB.16991.3, and PB.16991.4). All three novel transcripts were predicted to encode proteins with probability scores of 0.886, 0.888, and 0.876.

## Discussions and Conclusion

The brain has a unique transcriptome, characterized by extensive alternative splicing^37,42^. The NAc is of particular importance in reward and motivation and hence in the long-lasting effects of addiction^43^. To further the characterization of the NAc transcriptome, we applied long-read sequencing to NAc tissue pooled from cocaine-exposed and control mice in order to obtain a more complete picture of its isoform variation. In contrast to short-read sequencing, this approach produced more accurate isoform structures. These sequenced novel transcripts can complement the existing annotation for further studies.

Although the majority of the sequenced polyadenylated NAc isoforms were associated with known genes, a number of novel polyadenylated transcripts, both coding and non-coding, were identified. While the GENCODE annotation is an excellent resource for annotating the majority of NAc-derived transcripts, the BWMT annotation more accurately classified certain NAc-derived transcripts. This suggests that the BWMT transcriptome may be a useful complement to the GENCODE reference when seeking to annotate unknown murine RNAs. Interestingly, transcripts classified as intergenic exhibited a considerable expression range, suggesting that transcripts derived from intergenic regions can be heavily expressed despite originating from assumed non-genic stretches of the genome. This supports the presence of previously unannotated genes in mouse brain, which will require validation in future studies, prior to incorporating such novel genes into the standard murine transcriptome annotations. The current study validates several novel transcripts, both verifying splicing patterns present in the BWMT transcriptome, and validating the splicing patterns of novel NAc-specific transcripts.

Ontological analysis verified that the genes associated with highly expressed transcripts and novel transcript categories are enriched in NAc. When the novel categories were examined individually, the gene lists were associated with a range of cellular components. Interestingly, NNIC genes were significantly associated with regulation of GTP and regulation of synaptic plasticity, implicating the novel transcripts in regulating synapses within the NAc.

Previous studies have identified a series of addiction-related genes, which we examined in this study for the presence of novel transcripts. Cocaine- and amphetamine-regulated transcript (*Cartpt*), encoding the neuropeptide CART, may play a role in the actions of psychostimulant drugs of abuse, such as cocaine^44^. However, in this dataset, no novel transcripts were found to be associated with CARTPT. In contrast, FosB, a transcription factor implicated in the neuronal response to stress and drugs^38,39^, as well as its splice variant, ∆FosB, showed evidence of a 5′ truncation due to an alternative TSS^37^.

Interestingly, alignment of the amplicon sequences to the mm10 genome revealed that 5 of the 19 examined exon-exon junctions were derived from IAP repeats of the ERVK family (Supplementary File 2). A qRT-PCR analysis of the exon-exon junctions revealed that the IAP-derived amplicons are expressed at a similar level as other amplicons that were not generated from repeats (Supplementary Figure S3). Evolutionarily, ERVK elements are a relatively recent addition to the murine genome^45,46^. It is possible that IAPs and the ERVK family may be driving the formation of novel transcripts in the mouse brain. The current study does not extend to addressing the role of cocaine in modifying expression of novel transcripts. However, it is possible that cocaine administration may influence the expression of ERVK elements in neuronal tissues and by consequence the formation of novel transcripts.

In conclusion, the long read Iso-seq approach implemented in this study allowed the creation of a far more comprehensive NAc-derived transcriptome and the identification of NAc-specific transcripts. The NAc-derived transcriptome is intended for use as an annotation in future RNA-seq studies. In particular, the inclusion of both cocaine and saline samples allows this transcriptome to be a relevant reference for future addiction studies in mice.

## Methods

### RNA extraction

Ten 8–10 week old male C57BL/6 J mice were used to generate RNA for sequencing. Five of the mice were given intraperitoneal saline injections for 7 days, while the other 5 mice were given 20 mg/kg cocaine via intraperitoneal injection for 7 days. This approach (pooling tissue from saline- and cocaine-treated mice) was utilized to ensure that the captured transcriptome contained any splicing variants that might be induced only after cocaine exposure or conversely only expressed at baseline. NAc tissue was isolated by punch dissection one hour after the final injection and stored frozen at − 80 °C until RNA extraction. RNA from individual mice was extracted using Qiagen RNeasy Mini kit and pooled prior to library preparation. NAc was isolated from each mouse bilaterally, such that a total of 20 NAc 12 gauge punches were used for the experiment. Samples prepared for validation PCR were prepared in a similar fashion, with two minor exceptions. In the validation PCR samples, NAc tissue was isolated 24 h after the final injection, using 14 gauge punches. Further details on cocaine treatments and NAc dissections have been published previously^10^. All animal use was approved by Mount Sinai’s institutional animal care and use committee. All experiments involving live animals were performed in accordance with relevant guidelines and regulations. Additionally, all experiments involving live animals were performed in full compliance with ARRIVE guidelines 2.0 (https://arriveguidelines.org/).

### Iso-seq library preparation and sequencing

Briefly, two micrograms of high quality RNA (RIN = 8.8) was used as input into oligo-dT primed cDNA synthesis (Takara). Following double-stranded cDNA amplification, transcripts were size selected, separating transcripts < 4 kb from those > 4 kb by use of magnetic beads. The transcripts of each size bin were then equimolar pooled in order to adequately represent longer transcripts in the subsequent sequencing library. The pooled cDNA was used as input into SMRTbell library preparation as recommended by the manufacturer. The SMRTbell library was sequenced on a SMRTcell 8 M on the Sequel 2 platform using v2 chemistry within Mount Sinai’s Genomics Technology team.

### Bioinformatics analysis

Initial data processing was performed using the IsoSeq 3.1 software pipeline, which is incorporated into the online SMRTLink 8.0 bioinformatics tool suite (Pacific Biosciences). Intramolecular error correcting was performed using the circular consensus sequencing (CCS) algorithm to produce highly accurate (> Q20) CCS reads, each requiring a minimum of 3 polymerase passes. The polished CCS reads were then passed to the lima tool to remove IsoSeq and template-switching oligo sequences and orient the isoforms into the correct sense or antisense direction. The refine tool was then used to remove polyA tails and concatemers from the full-length reads and finally, the cluster algorithm was invoked to perform a reference-free clustering of the sequences into final consensus isoforms ready for downstream analysis.

The resulting FASTA data of high quality (HQ), polished isoforms were used in the following downstream analysis. The HQ isoforms were aligned to the GRCm38 *Mus musculus* genome assembly using the splice-aware aligner, GMAP^47^ (version 2019–02-15). All uniquely-mapped isoforms were used as input into the TAMA Exon Cascade Collapse algorithm (version 2019–11-19) to reduce isoform redundancy. The cDNA_Cupcake^12^ (version 11.0.0) tool suite was then used to extract full-length counts for the collapsed isoforms. Next, the SQANTI2 (version 7.4.0) tool suite was used to remove artifactual transcripts with evidence of RT-switching and genomic polyA intra-priming errors. Using SQANTI2^12–14^, the final list of uniquely-mapped, collapsed and filtered isoforms were characterized against the M25 release of GENCODE gene annotations. The collapsed, unique isoforms were also annotated with MatchAnnot (version 2015-02-18)^16^, using the mm10 GENCODE annotation (release M25). An in-house pipeline to automatically perform the above steps is available at https://github.com/shenlab-sinai/Isoseq_processing. Coding potential for unique transcripts was calculated using COME (COding potential from Multiple fEatures)^48^, with the pre-calculated murine (mm10) model^49^.

The Brain-enriched Whole Mouse Transcriptome (BWMT) was derived from three individual short-read RNA-seq studies, two Pacbio RNA-seq studies, and the NONCODE database. The first study, GSE125483, assessed a variety of mouse tissues (Adrenal, Colon, Heart, Liver, Lung, Muscle, Pituitary, Skin, Thyroid and Brain)^20^. The second study, GSE107423, assessed healthy brain tissue^21^. The third study, GSE112348, extracted tissue from Cortex^22^. GTF files from the three studies were extracted from the GEO database. The two Pacbio long-read technology studies, GSE93848 and GSE138760, were designed to capture long-read transcriptome of various murine tissues (including brain)^23^, and preimplantation embryos^24^, respectively. The NONCODE database (version v6.0) for mouse contains 131,974 long non-coding transcripts^25,26^.

NAc-derived short reads from previously published short read (100 bp) libraries^10^ (GSE42805) were subjected to de novo transcriptome assembly, using SPAdes (version 3.14.1). The de novo fasta file was collapsed using the TAMA Exon Cascade Collapse algorithm and converted to a GTF file. This GTF file was then processed with the ‘cuffmerge’ command available in Cufflinks (version 2.2.1), using the GENCODE M25 gene annotation as a reference, producing the de novo short-read NAc transcriptome. To produce the BWMT annotation, GTF files from the three studies (GSE125483, GSE107423, and GSE112348) were combined using the ‘cuffmerge’ command available in Cufflinks (version 2.2.1), using the GENCODE M25 gene annotation as a reference.

Expression of the isoforms derived from the Iso-seq dataset was assessed using previously published short read (100 bp) libraries (GSE42805)^10^. Expression was calculated with Kallisto (version 0.46.2), in conjunction with the GTF file generated from the Iso-seq processing pipeline, for all six short-read RNA-seq samples from the NAc of three replicates injected with cocaine (SRR629622, SRR629623, SRR629624) and three control replicates (SRR629625, SRR629626, SRR629627) to the mm10 genome. Normalized read counts were presented as Transcripts Per Million (TPM).

Ontological analysis of gene sets was performed in R with enrichR (version 2.1). The “Mouse_Gene_Atlas”, “GO_Molecular_Function_2015”, “GO_Cellular_Component_2015”, and “GO_Biological_Process_2015” databases were used as ontological databases for calculating enrichment of gene sets. A maximum adjusted *P*-value threshold of 0.05 was used as a cutoff for consideration of GO terms whenever possible.

### Validation PCR

Exon-exon primers were designed and utilized for novel transcript validation. For qPCR validation, RNA extracted from NAc of four saline- and five cocaine-treated mice were separately converted to cDNA with iScript (Bio-Rad) with a 500 ng RNA input. 100 ng per sample was combined for a total of 1000 ng RNA input. Real-time qPCR was performed using SybrGreen Fast master mix and standard cycling conditions on a QuantStudio 5 and analyzed by the 2^−∆Ct^ method with *ActB* as a control gene.

For TA cloning, amplicon of each primer set was obtained through PCR using Platinum Taq DNA Polymerase High Fidelity (ThermoFisher) and cleaned up using NucleoSpin Gel and PCR Clean-Up (Macherey–Nagel). TA cloning of the amplicons was accomplished by pGEM-T Easy Vector Systems (Promega), and plasmid were extracted through NucleoSpin Plasmid (Macherey–Nagel). To acquire DNA sequences of amplicons, plasmids were subjected to Sanger sequencing with T7 primer (5′- TAATACGACTCACTATAGGG-3′).

## Supplementary Information

Supplementary Information 1.

Supplementary Information 2.

Supplementary Information 3.

Supplementary Information 4.

Supplementary Information 5.

Supplementary Information 6.

Supplementary Information 7.

## Data Availability

The dataset described in this study is available at GSE129462.

## Code availability

An in-house pipeline to perform the majority of processing steps is available at https://github.com/shenlab-sinai/Isoseq_processing.
