# Supplementary material for: Long read, isoform aware sequencing of mouse nucleus accumbens after chronic cocaine treatment
Source: Sci Rep. 2021 Mar 24;11:6729. doi: 10.1038/s41598-021-86068-7 (PMC7991652; doi:10.1038/s41598-021-86068-7)

Title: **Long read, isoform aware sequencing of mouse nucleus accumbens after chronic cocaine treatment**

Molly Estill^1^, Efrain Ribeiro^1^, Nancy J. Francoeur^2^, Melissa L. Smith^2^, Robert Sebra^2,3^, Szu-Ying Yeh^1^, Ashley M. Cunningham^1^, Eric J. Nestler^1^, and Li Shen^1^

^1^Nash Family Department of Neuroscience and Friedman Brain Institute, ^2^Department of Genetics and Genomic Sciences and Icahn Institute for Data Science and Genomics Technology, Icahn School of Medicine at Mount Sinai, New York, NY 10029 and ^3^­­Sema4, a Mount Sinai venture, Stamford CT, USA

*correspondence: [li.shen@mssm.edu](mailto:li.shen@mssm.edu)

**Supplementary Data**

**Supplementary File 1. SQANTI2 classification of transcripts, using GENCODE as reference annotation.**

**Supplementary File 2. PCR validation of select novel transcripts.**

**Supplementary File 3. BWMT annotation**

**Supplementary File 4. SQANTI2 classification of transcripts, using BWMT annotation as reference annotation.**

**Supplementary File 5. *De novo* short-read NAc annotation**

**Supplementary File 6. Ontological enrichment of novel transcripts.**

**Supplementary Fig. S1. SQANTI classification**. (A) Multi-exonic intergenic transcript PB.15060.1, colored in dark blue, adjacent to the VARS (valyl-tRNA synthetase) gene. (B) Antisense transcript PB.9606.1, colored in dark blue, antisense to the DNAJB12 gene*.* (C) Genic Genomic transcript PB.16618.1, shown in dark blue, overlapping the RTL8C gene. (D) Fusion transcript PB.5170.6, a fusion between the genes SSC4D and YWHAG. Exons obtained from YWHAG are colored in green, while the exon obtained from SSC4D is colored in dark blue (E) Novel Not in Category transcript, PB.16873.5, originating from *Plp1*. The novel 5’ segment is colored in light blue. In all panels, the novel transcript of interest is highlighted in a pale red box. In each panel, the top track represents the GENCODE annotation, the middle track represents the gene models generated from the Iso-seq pipeline, and the bottom track represents the unique Iso-seq reads. Note that each unique Iso-seq read is supported by two or more full-length reads.


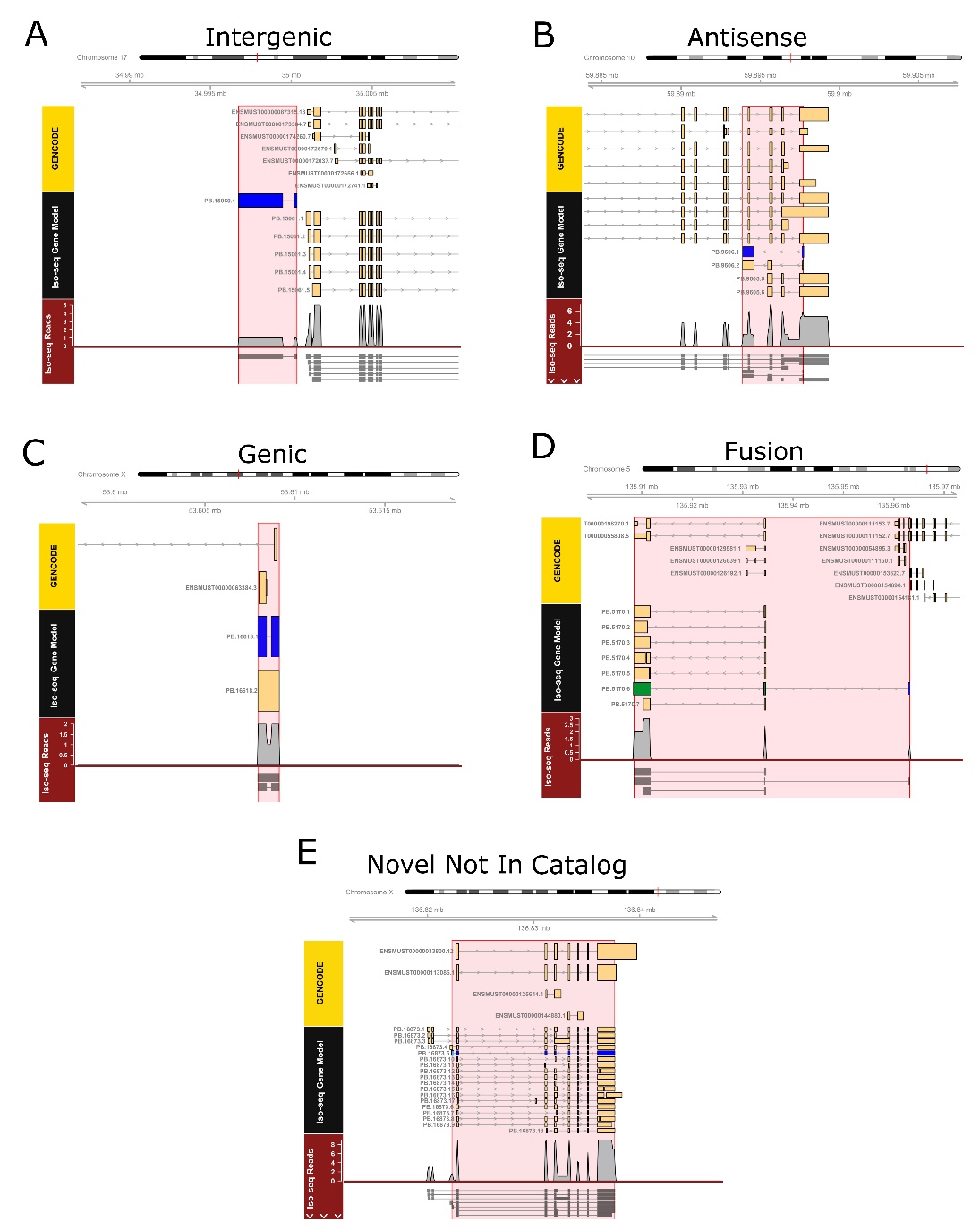


**Supplementary Fig. S2. MatchAnnot score across SQANTI2 classifications.** FSM = Full Splice Match; ISM = Incomplete Splice Match; NIC = Not In Catalog; NNIC = Novel Not In Catalog.


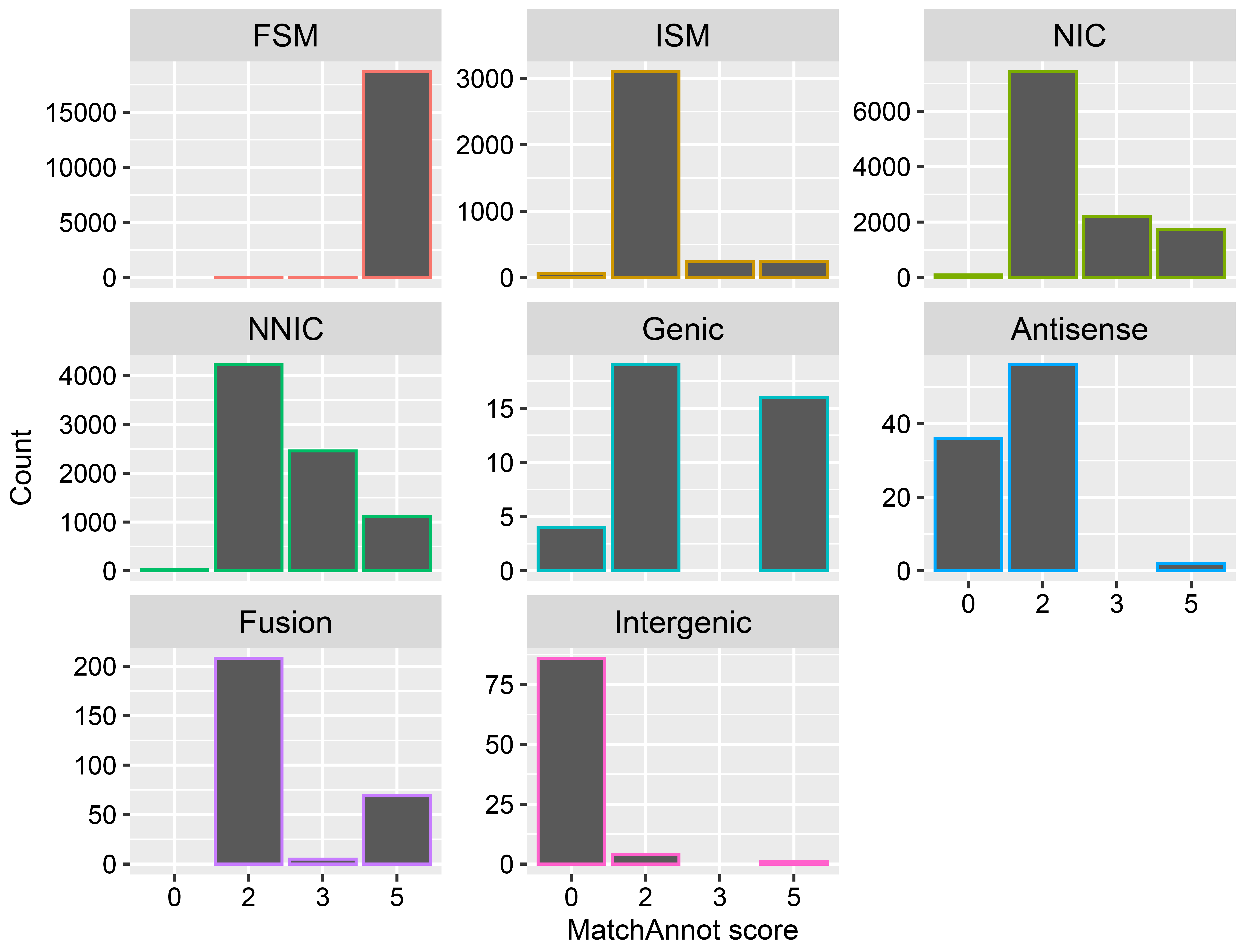


**Supplementary Fig. S3. qRT-PCR quantification of novel transcripts.** The Y-axis indicates the 2^−∆Ct^ quantification with *ActB* as a control gene. For each transcript, the individual samples are shown as black points. The mean of each transcript is shown as a red point, with the standard deviation shown as red bars. Transcripts marked with black stars indicate transcripts whose amplicon is derived from an ERVK repeat.

**
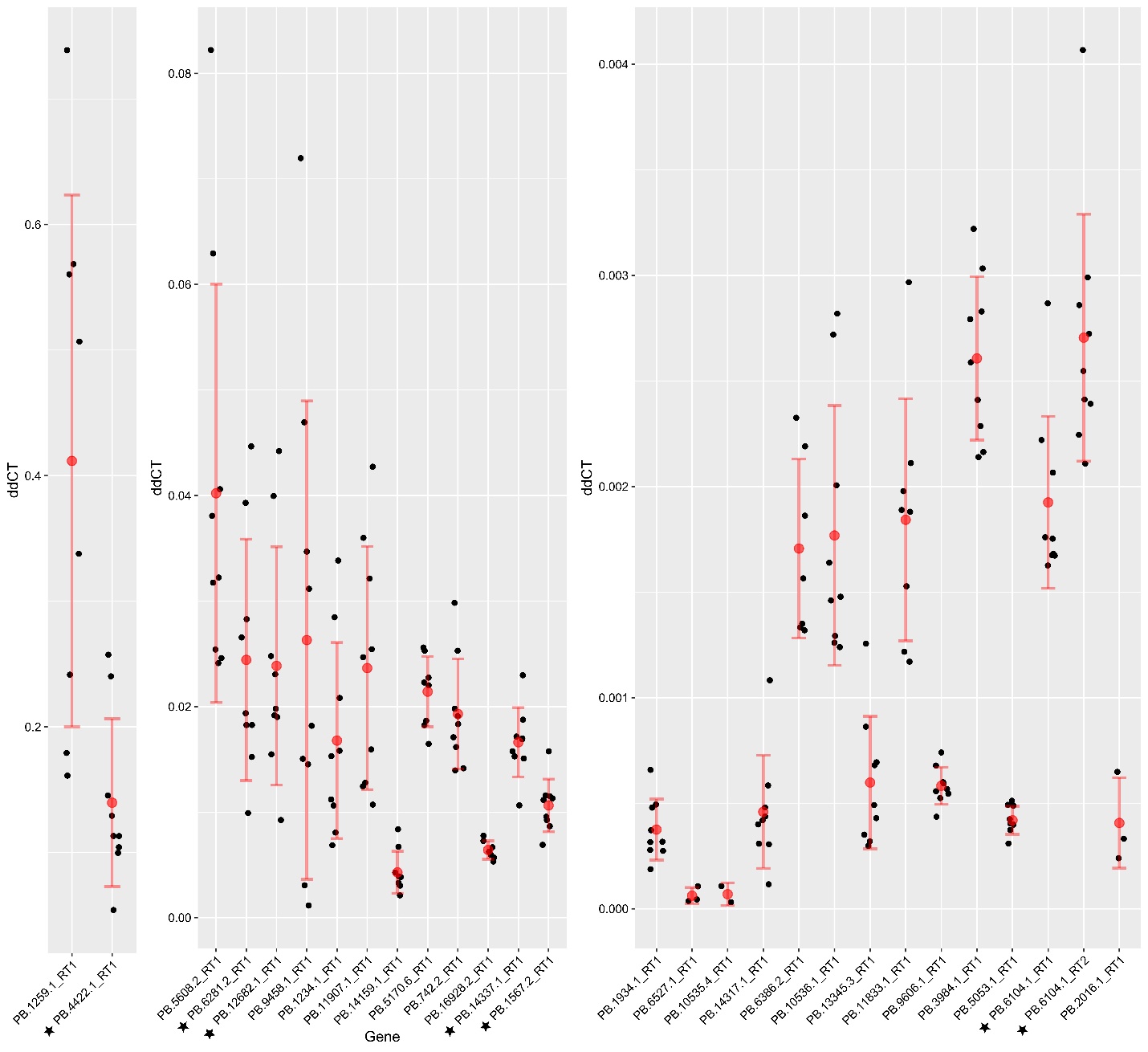
**

**Supplementary Fig. S4. GFAP expression across short-read and Iso-seq libraries.**

The top track represents the GENCODE annotation, the second track represents the gene models generated from the Iso-seq pipeline, and the third track represents the unique Iso-seq reads. The bottom two tracks represent the short read coverage in NAc from mice injected with saline or cocaine. GFAP is highlighted in a pale red box. Note that each unique Iso-seq read is supported by two or more full-length reads.


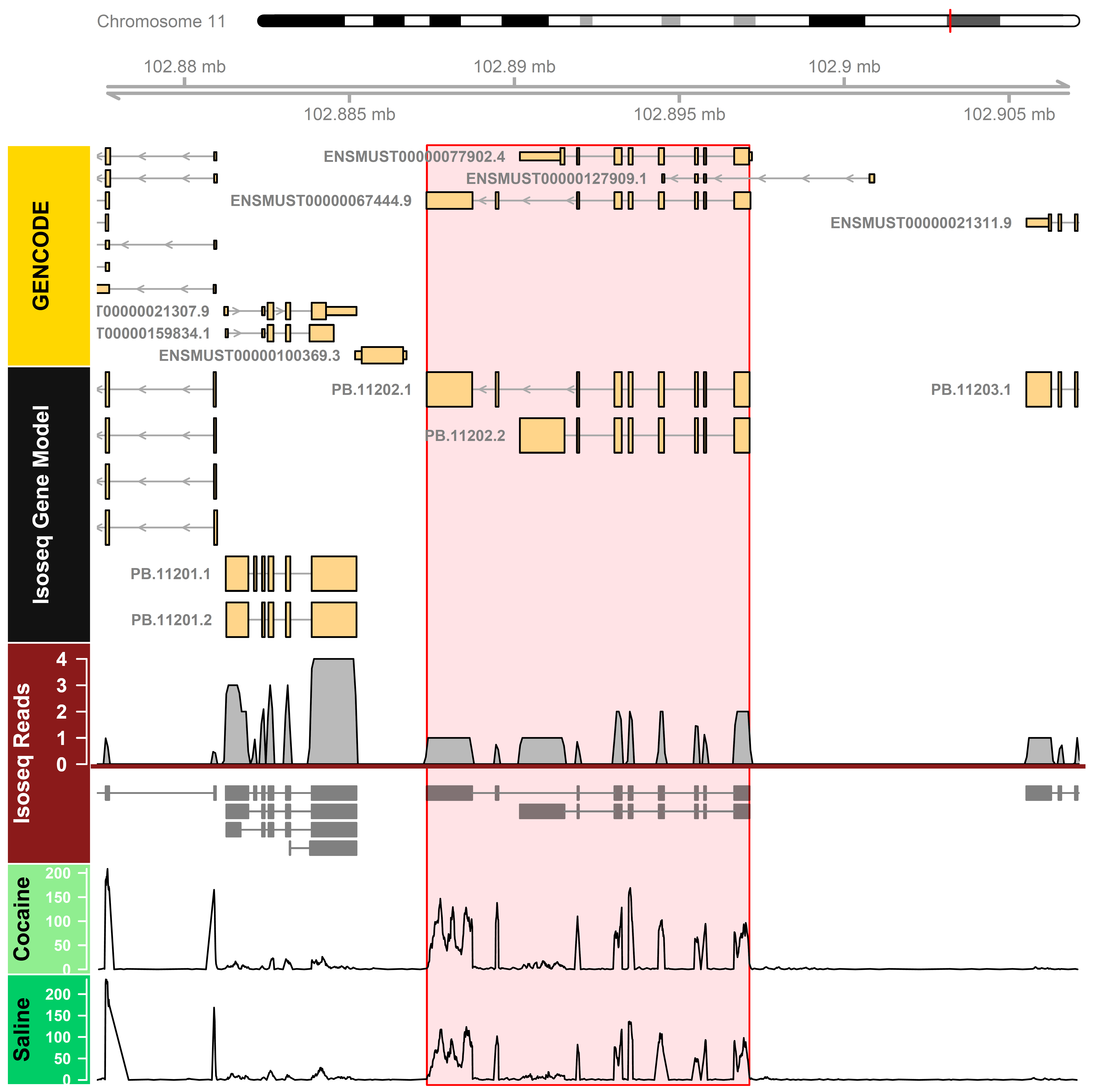


**Supplementary Fig. S5. Distribution of transcript expressions in short-read RNA.**

Box and whisker plots of the mean expression value across 6 short-read NAc samples. The Y-axis indicates the log10-transformed TPM, with 1 TPM added to prevent undefined values.

Among the different SQANTI2 categories, certain categories, such as FSM and ISM, were associated with slightly higher expression values than the others. FSM = Full Splice Match; ISM = Incomplete Splice Match; NIC = Not In Catalog; NNIC = Novel Not In Catalog.


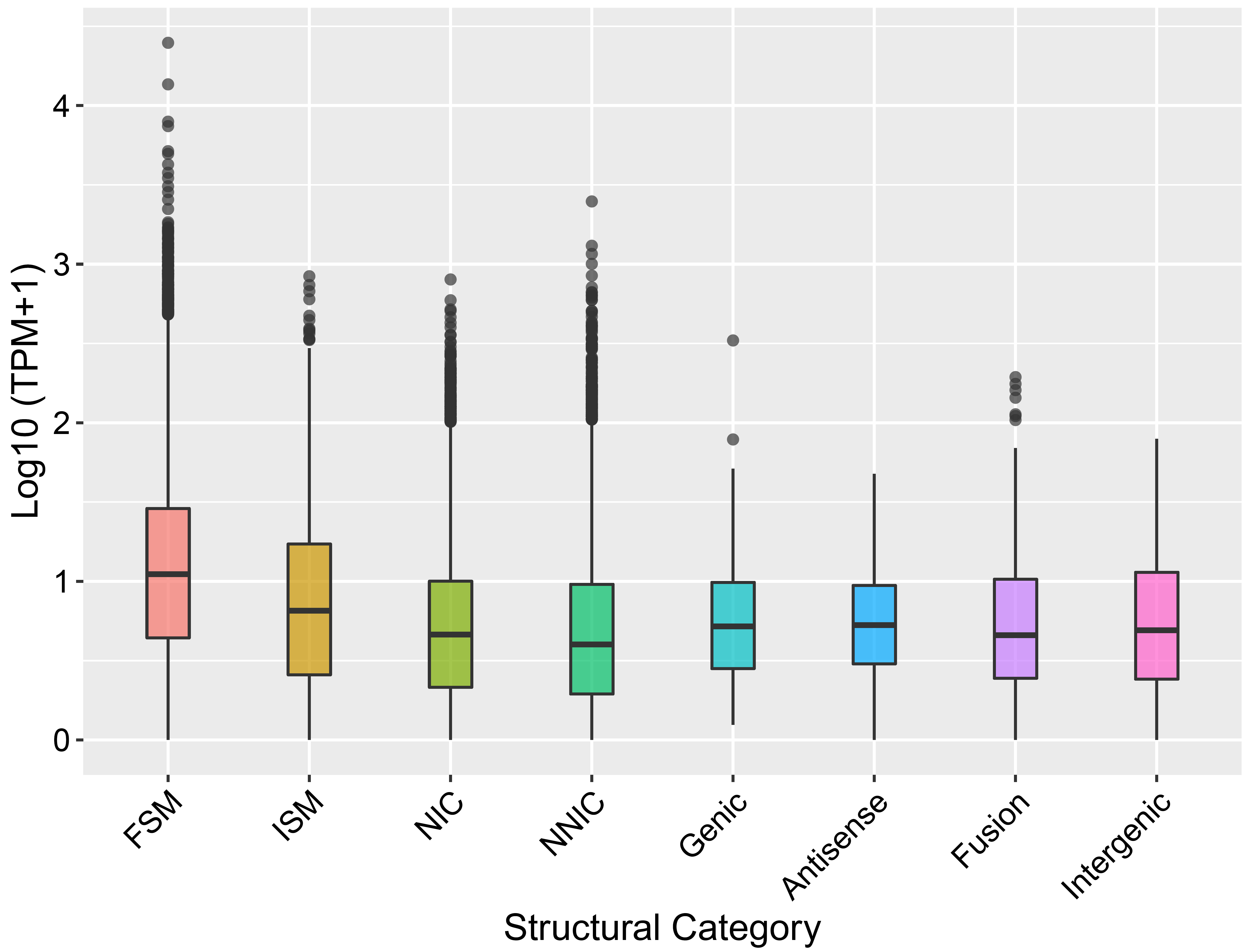

Supplement: Supplementary file 1 — Supplementary Information 1. [file 41598_2021_86068_MOESM1_ESM.docx]
